# Comparing Transcriptome and Stem Anatomy Analysis Reveals That the Phenylpropanoid Pathway Is a Key Driving Factor for Lodging Resistance in *Brassica rapa*

**DOI:** 10.3390/plants15071134

**Published:** 2026-04-07

**Authors:** Hongyan Wei, Junmei Cui, Jiaping Wei, Yan Fang, Zefeng Wu, Guoqiang Zheng, Zigang Liu

**Affiliations:** State Key Laboratory of Aridland Crop Science, Gansu Agricultural University, Lanzhou 730070, China; weihy@st.gsau.edu.cn (H.W.); cuijm@gsau.edu.cn (J.C.); weijp@gsau.edu.cn (J.W.); fangy@gsau.edu.cn (Y.F.); wuzf@gsau.edu.cn (Z.W.); zhenggq@st.gsau.edu.cn (G.Z.)

**Keywords:** rapeseed, lodging, agronomic traits, stem, transcriptome

## Abstract

*Brassica rapa* is widely cultivated in alpine and cold mountainous regions due to its strong cold tolerance. However, lodging severely limits its yield and quality. This study integrated agronomic traits, stem microstructure, and transcriptomic profiles to explore the mechanism of lodging resistance by comparing a resistant cultivar (Ganyou 3064, GY) and a susceptible cultivar (Tianyou 2022, TY) across four developmental stages (full flowering, final flowering, podding, and maturity). At the four growth stages, the stem breaking strength of GY was 1.71, 1.93, 1.88, and 1.88 times that of TY, respectively. Compared with TY, the gravity center height of GY was decreased by 25.04%, 16.6%, 11.18%, and 8.98% at these four stages, respectively. Similarly, the lodging index of GY was decreased by 65.94%, 55.08%, 56.06%, and 55.63% compared with TY, respectively. Biochemical and anatomical analyses revealed that compared with TY, the lignin content of GY increased by 1.93%, 2.7%, 3.05%, and 3.42% at the four stages, while the cellulose content increased by 92.75%, 45.32%, 44.4%, and 49.92%, respectively. Meanwhile, the epidermal thickness, cortical thickness, vascular bundle length, vascular bundle area, and vascular bundle density of GY were also significantly increased. Transcriptomic and KEGG pathway analyses revealed a predictive defense mechanism of GY. At the final flowering stage, GY showed pre-activation of hormone and MAPK signal transduction, as well as phenylpropanoid biosynthesis; it shifted to energy supply and sustained cell wall reinforcement at the podding stage. In addition, upregulated genes in phenylpropanoid biosynthesis (such as *PAL3*, *CCoAOMT*, and *CAD9*) indicated that enhanced stem lignification is a key molecular determinant of lodging resistance. In summary, GY enhances its lodging resistance through coordinated morphological and transcriptional regulation. This study is the first to integrate the lodging characteristics of *Brassica rapa*, offering valuable candidate genes and phenotypic markers for molecular breeding.

## 1. Introduction

Rapeseed is the third-largest oil crop globally, playing important roles in vegetable oil supply and economic development [1]. However, lodging causes significant reductions in the yield and seed oil content of rapeseed. Compared with normally growing plants, lodging-susceptible rapeseed varieties typically exhibit a yield reduction of 10–30%, which can even exceed 50% under severe lodging conditions, accompanied by a concurrent decrease in seed oil content of 10–30% [2]. On the other hand, lodging severely impedes mechanical harvesting, necessitating manual cutting, thereby drastically reducing production efficiency and economic benefits. Lodging not only affects its value as an edible oil source but also significantly reduces the economic benefits of rapeseed production [3,4]. Thereby enhancing lodging resistance has become a key goal in breeding programs, critical for ensuring stable agricultural production and providing high-quality raw materials.

To address the significant challenge of lodging in rapeseed, numerous studies have been conducted to investigate the mechanisms underlying lodging. Rapeseed lodging is a complex agronomic trait with five major inducing factors, namely the inherent characteristics of varieties, fertilization regime, sowing density, field management practices, and precipitation, among which the inherent characteristics of varieties represent the intrinsic core determinant of lodging resistance [5,6,7].

At the agronomic trait level, the synergistic effect of favorable traits such as moderate plant height, superior stem strength, robust and well-developed root systems, and rational plant architecture can significantly enhance the lodging resistance of plants [6,8,9]. Excessive plant height in rapeseed leads to lodging, which can reduce rapeseed yield by more than 60% [9]. Therefore, semi-dwarf hybrid rapeseed varieties have been developed to improve lodging resistance [10].

At the physiological level, stem mechanical strength is recognized as the core determinant. Cellulose, hemicellulose, and lignin are the main components of the plant cell secondary wall, and their content levels are key determinants of stem mechanical strength. Lignin content serves as a key indicator for lodging resistance [11,12,13,14,15,16]. A number of enzymes involved in lignin biosynthesis have been reported to promote lignin accumulation and thus enhance plant lodging resistance [17,18]. Moreover, *BnaMYB43* can enhance the degree of vascular lignification, thereby improving lodging resistance in rapeseed [19].

At the molecular level, genome-wide association study (GWAS) and transcriptomic analyses have identified critical genetic loci and candidate genes regulating lignin biosynthesis [20]. Population-based studies have identified SNPs significantly associated with resistance to lodging, stem mechanical strength, and lodging coefficient, as well as genes involved in the regulation of lignin biosynthesis in rapeseed [20,21,22,23]. By integrating transcriptome expression analysis and GWAS, Wei et al. identified four candidate genes regulating lignin biosynthesis [20]. By integrating GWAS and gene co-expression network analysis, Li et al. prioritized several promising candidate genes, including *ESK1*, *CESA6*, and *FRA8* [24]. Studies on transcription factors demonstrated that downregulating the expression of *BnMYB69* in *Brassica napus* significantly affects lignin and cellulose biosynthesis in stems [25], whereas *BnMYB43* positively regulates vascular bundle lignification, thereby enhancing lodging resistance in rapeseed [19]. Furthermore, studies in soybean have demonstrated that the gibberellin signaling pathway modulates lodging resistance by controlling plant height. RIN1 inhibits internode elongation through the STF1/STF2-GA2ox7 regulatory pathway by mediating gibberellin metabolism, thus reducing the risk of lodging [26].

Recent advances in understanding rapeseed lodging resistance mechanisms have revealed multi-dimensional regulatory networks involving physiological structure, molecular genetics, and environmental interactions. However, certain limitations still exist. For instance, research mainly focus on aboveground parts, with a lack of studies on root systems and microstructures. Although numerous key potential genes associated with lodging resistance have been identified in rapeseed, the detailed functions and molecular mechanisms of most of these genes remain largely uncharacterized. Furthermore, current researches on rapeseed lodging resistance have mainly focused on *Brassica napus* (allotetraploid), with relatively limited research on diploid *Brassica rapa*. *Brassica rapa* is widely cultivated in alpine and cold mountainous regions due to its strong cold tolerance. Its low cultivation cost and adaptability to marginal lands further underscore its potential in sustainable agriculture and ecological remediation. Therefore, systematically investigating the lodging resistance mechanisms of *Brassica rapais* of great theoretical and practical significance for breeding lodging-resistant *Brassica rapa* varieties. To address these research gaps, this study provides a comprehensive insight into the lodging resistance mechanism of *Brassica rapa* by integrating phenotypic, root-system-related, mechanical, histochemical, and transcriptomic analyses. The findings are expected to provide a valuable theoretical foundation and candidate gene resources for breeding high-yielding, lodging-resistant rapeseed varieties.

## 2. Results

### 2.1. Lodging Phenotype, Rapeseed Yield and Varietal Differences Between Two Rapeseed Varieties

**Figure 1:**
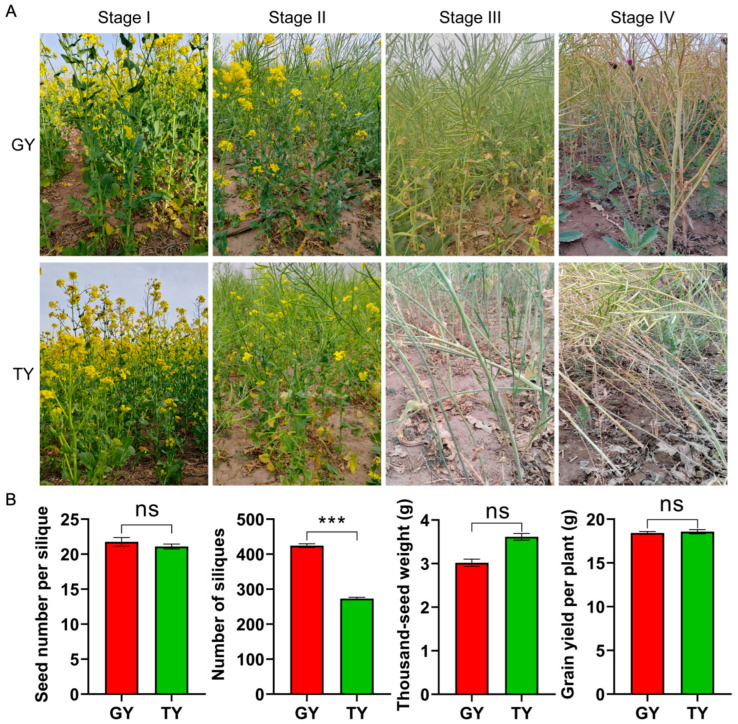
Lodging phenotypes and yield trait quantification of two rapeseed varieties. (**A**) Growth phenotypes of the lodging-resistant cultivar Gan You 3064 (GY) and the lodging-susceptible cultivar Tian You 2022 (TY) across four developmental stages (Stage I: full flowering, Stage II: final flowering, Stage III: podding, Stage IV: maturity). (**B**) Analysis of yield components: seeds per silique, silique number per plant, thousand-seed weight, and total yield per plant. Data represent mean ± SD (*n* = 10). One-way analysis of variance (ANOVA) was used. Asterisks (***) indicate significant differences at the *p* < 0.001 level, and “ns” represents no significant difference.

To clarify the lodging resistance and potential for production application of rapeseed varieties, this study conducted dynamic monitoring of lodging under field conditions for two cultivars, Gan You 3064 (GY) and Tian You 2022 (TY). The results, shown in Figure 1A, revealed significant differences in lodging resistance between the two varieties. At full flowering stage (Stage I) and final flowering stage (Stage II), stems of both cultivars remained upright with no signs of lodging. By podding stage (Stage III), the majority of TY plants exhibited stem bending, with a minority showing stem breakage, whereas all GY plants remained upright. At maturity stage (Stage IV), most TY plants suffered from stem breakage, while GY plants continued to maintain an upright posture. In summary, TY was identified as a lodging-susceptible variety, primarily exhibiting stem lodging. In contrast, GY demonstrated exceptional lodging resistance, which validated its superior potential for practical field applications.

Statistical analysis of key yield-related agronomic traits indicated that the two varieties exhibited complementary characteristics in yield formation, with no significant difference in overall yield per plant (Figure 1B). Specifically, GY had a significantly higher number of siliques than TY, indicating a distinct advantage in the number of seed-bearing organs. In contrast, TY showed a higher thousand-seed weight, reflecting its advantage in individual seed mass. Notably, no significant differences were observed in the number of seeds per silique or in the final yield per plant between the two cultivars. These results suggest that the two varieties achieve similar overall yield levels through complementary contributions of different yield-related traits.

Analysis of 14 key quality traits in the seeds of the two varieties revealed significant differences in five indicators: oil content, erucic acid, glucosinolates, oleic acid, and linoleic acid (Table 1). The oil content of TY was 2.879% higher than that of GY. But TY performed poorly in several core quality parameters for edible oil. Specifically, GY exhibited significantly higher oleic acid (42.82% vs. 29.52%) and linoleic acid (15.60% vs. 12.76%) contents, alongside substantially lower levels of anti-nutritional factors including erucic acid (23.12% vs. 39.74%) and glucosinolates (67.34 μmol/g vs. 89.17 μmol/g). These differences position GY as a more desirable candidate for edible oil production. Oils with low erucic acid, high oleic acid, and high linoleic acid can protect cardiovascular health, reduce bad cholesterol, and supplement essential fatty acids for the human body. GY, despite its slightly lower oil content, exhibited higher levels of beneficial fatty acids and lower levels of anti-nutritional factors. Therefore, GY is a more suitable vegetable oil for consumption compared to TY.

### 2.2. Comparison of Lodging-Related Agronomic Traits in Aerial Parts

**Figure 2:**
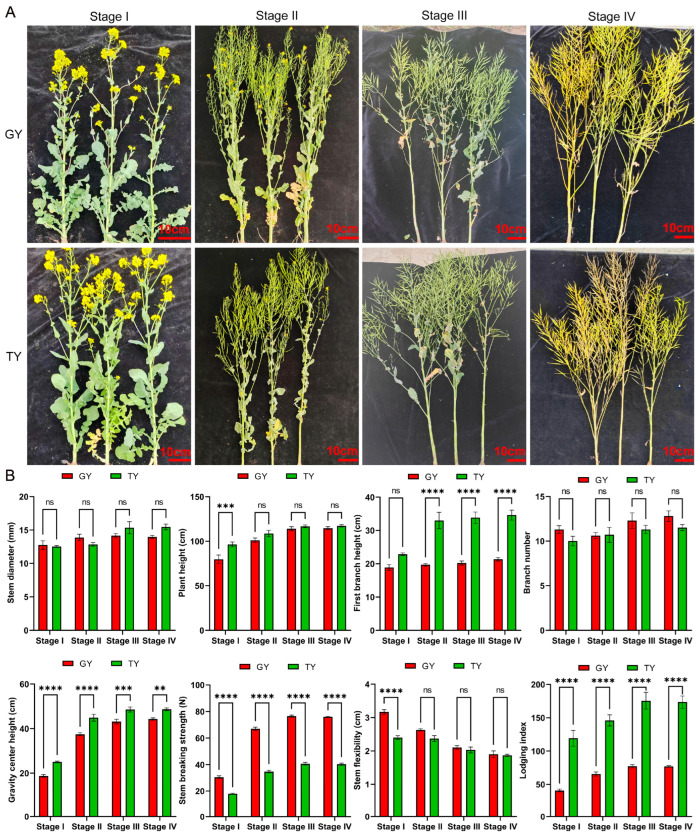
Evaluation of lodging-related aerial traits in two rapeseed varieties. (**A**) Representative images of the aerial morphology of the two varieties. The scale bar represents 10 cm. (**B**) Quantitative comparison of lodging-related traits, including stem diameter, plant height, first branching height, branch number, center of gravity height, stem breaking strength, stem flexibility, and lodging index. Data are presented as mean ± SD (*n* = 10). Two-way ANOVA analysis was used. Asterisks denote significance levels: ** *p* < 0.01, *** *p* < 0.001, and **** *p* < 0.0001. “ns” denotes no significant difference.

Phenotypic performance of the aerial parts is illustrated in Figure 2A. Significant differences in lodging-related aerial traits were observed between the two materials (Figure 2B). Specifically, GY demonstrated significantly greater stem breaking resistance across all four stages, as well as significantly higher stem flexibility at full flowering stage. In contrast, the center of gravity height and lodging index of GY were significantly lower than those of TY throughout all stages. The first branch height of GY was also significantly lower than that of TY from final flowering stage to maturity stage. Although TY exhibited greater plant height than GY across all stages, a significant difference was only observed at full flowering stage. No significant differences were detected in stem diameter or branch number between the two materials.

Given that lodging typically occurs at podding stage, the observed differences in first branch height, center of gravity, stem breaking resistance, and lodging index represent key factors contributing to the variation in lodging resistance between the two varieties.

### 2.3. Comparison of Above- and Below-Ground Biomass Allocation and Root Architecture

**Figure 3:**
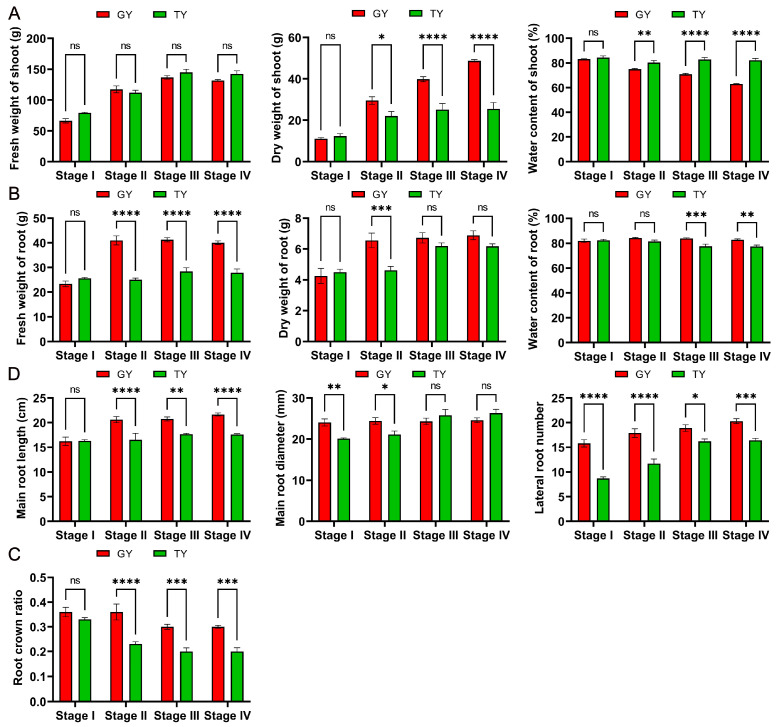
Comprehensive biomass and root system profiling. Biomass metrics for the aerial (**A**) and underground (**B**) parts, including fresh weight, dry weight, and moisture content. (**C**) The root crown ratio, calculated based on fresh weight. (**D**) Root system architecture traits, comprising total root length, root diameter, and the number of lateral roots. Data are presented as mean ± SD (*n* = 10). Two-way ANOVA analysis was used. Asterisks denote significance levels: * *p* < 0.05, ** *p* < 0.01, *** *p* < 0.001, and **** *p* < 0.0001. “ns” denotes no significant difference.

As shown in Figure 3A, no significant differences were observed in the fresh weight of the above-ground parts between GY and TY across the four growth stages. In contrast, the dry weight of GY was significantly higher than that of TY in the last three stages, although no notable difference was found at the full flowering stage. At maturity stage, the dry weight of GY was 1.91 times greater than that of TY. Additionally, the water content of GY exhibited a decreasing trend throughout the developmental stages and was significantly lower than that of TY, with the most pronounced difference observed at maturity stage, where GY showed a 1.3-fold lower value than TY. These results indicate that GY accumulated more dry biomass.

As illustrated in Figure 3B, the root fresh weight of GY was significantly greater than that of TY at all stages except the full flowering stage. Both GY and TY exhibited an increase in root dry weight over time, with GY consistently displaying higher values; however, a statistically significant difference was only detected at the final flowering stage. At this stage, the differences in root fresh weight and root dry weight were most pronounced, with GY being 1.64 times and 1.42 times higher than TY, respectively. The root water content of both materials decreased during development, and GY had significantly higher root water content than TY at the podding stage and maturity stage.

Based on the above- and below-ground biomass data, the root crown ratio was calculated. As shown in Figure 3C, the root crown ratio of both materials decreased with progression of the growth stages. Except at the full flowering stage, GY consistently showed a significantly higher root crown ratio than TY.

As shown in Figure 3D, from the full flowering stage to maturity stage, the root length of GY gradually increased, with the most pronounced growth occurring between the full and final flowering stages, showing a 27% increase compared to the initial value at full flowering. In contrast, TY maintained relatively stable root length with no clear changes across the four stages. Except at the full flowering stage, GY exhibited significantly greater root length than TY throughout the developmental period. Regarding root diameter, GY showed significantly thicker roots than TY at all stages except the full and final flowering stages; however, no significant differences were observed in the later stages. In terms of lateral roots, GY consistently produced a significantly higher number of lateral roots than TY across all stages. The most substantial differences in root diameter and lateral root number between the two materials were observed at the full flowering stage, where GY exceeded TY by 1.2-fold and 1.82-fold, respectively.

### 2.4. Gene Differential Expression Analysis

**Figure 4:**
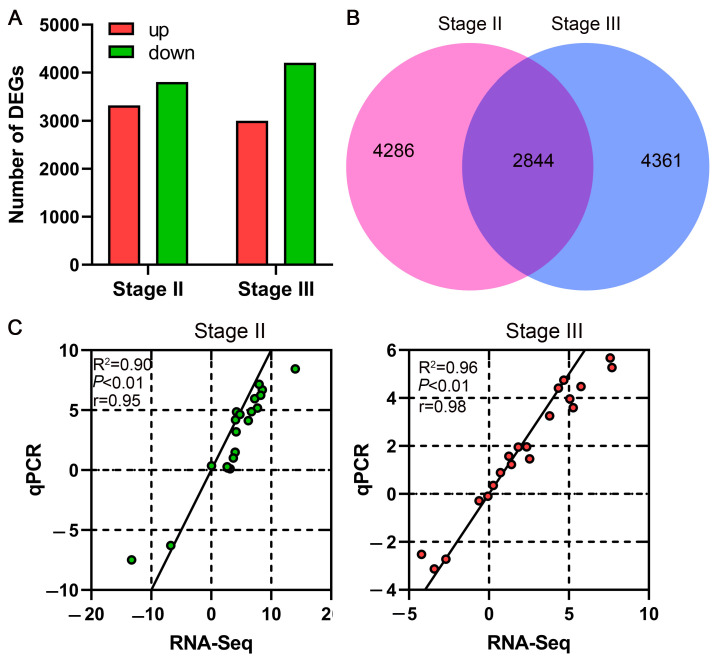
Differentially expressed genes (DEGs). (**A**) DEGs in GY compared to TY at the final flowering (Stage II) and podding (Stage III) stages. (**B**) Venn diagram of DEGs. The numbers in the pink and blue circles represent the numbers of DEGs of GY relative to TY at the final flowering stage (Stage II) and podding stage (Stage III), respectively. The overlapping regions of the circles indicate the number of shared DEGs between the two stages. (**C**) Correlation between qPCR and RNA-seq data. Scatter plot of the log2 fold change from RNA-seq versus the relative fold change from qPCR for 20 representative genes (including up-regulated, down-regulated, and non-significant genes). The R^2^ value was calculated using Pearson correlation analysis, indicating consistency between qPCR and RNA-seq data.

Given that stem bending in genotype TY occurs during the podding stage, we conducted transcriptomic analyses of stem tissues both before and after the bending event. This comparative approach was designed to identify transcriptomic differences between the two varieties that may underlie the divergence in lodging resistance. A total of 11,491 differentially expressed genes (DEGs) were identified between the two comparison groups. At the final flowering stage, 3321 genes were up-regulated and 3809 were down-regulated in GY material relative to TY. At the podding stage, 3000 genes were up-regulated and 4205 were down-regulated (Figure 4A). A core set of 2844 DEGs was common to both stages, highlighting consistently divergent regulatory programs (Figure 4B). A subset of 20 genes, comprising significantly up-regulated, significantly down-regulated, and non-significantly altered genes, was subjected to qPCR validation (Figure S1). The results demonstrated a significant correlation with the transcriptomic data, with a correlation coefficient (R2) over 0.9, thereby validating the accuracy and reliability of the RNA-seq findings.

**Figure 5:**
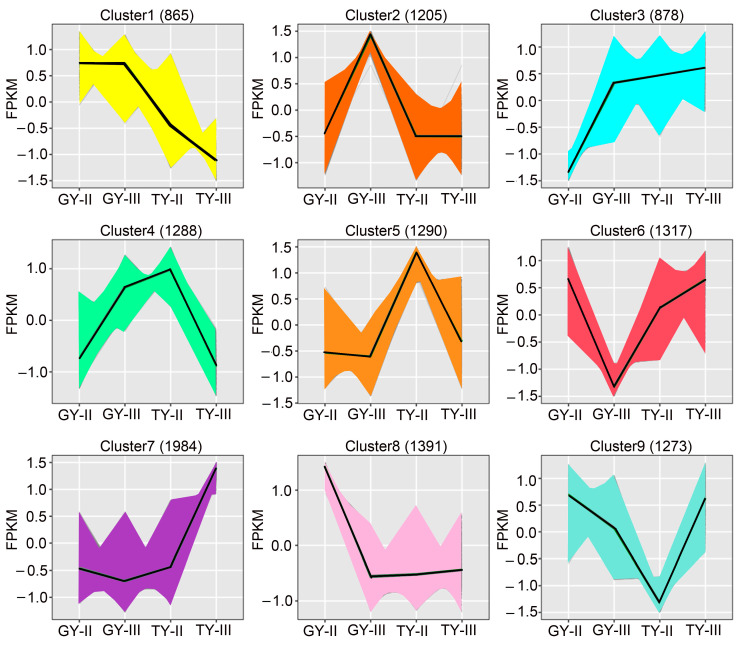
Cluster analysis of DEGs by K-means algorithm.

To investigate the expression patterns of DEGs identified in the two cultivars, we performed K-means clustering analysis, which enabled the identification of gene clusters with distinct expression profiles. A total of 11,491 DEGs were grouped into nine clusters, each containing between 865 and 1984 genes with similar expression trends (Figure 5). Clusters 1 and 2, comprising 865 and 1205 DEGs, respectively, exhibited high expression in the lodging resistant genotype GY. In contrast, clusters 3, 5, and 7, containing 878, 1290, and 1984 DEGs, respectively, were down expressions in GY.

### 2.5. KEGG Pathway Analysis

**Figure 6:**
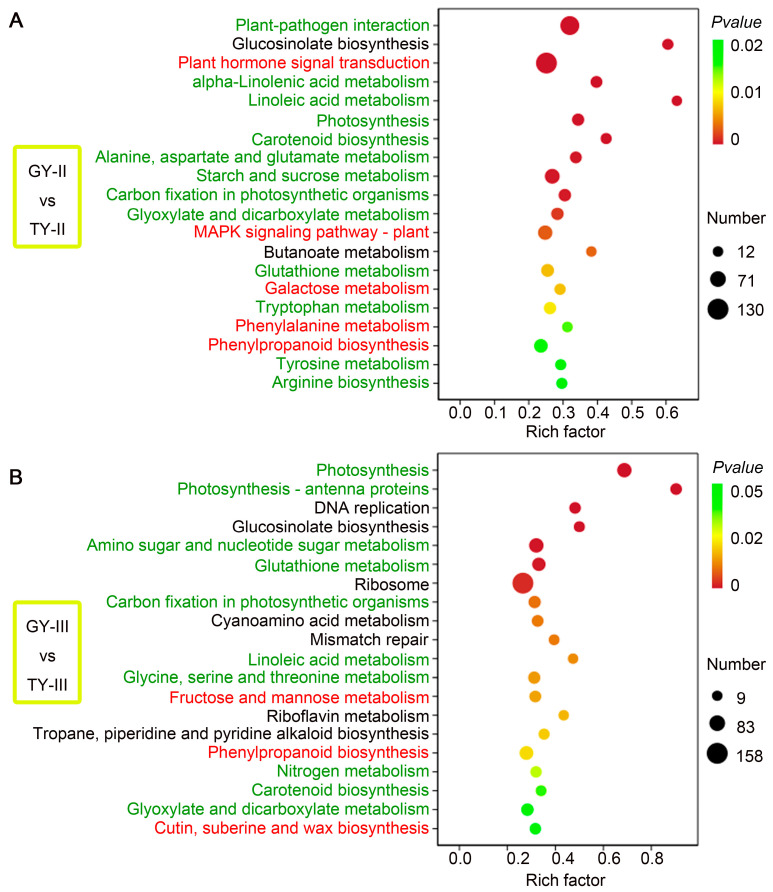
KEGG analysis. The top 20 KEGG enriched pathways of DEGs in GY compared with TY at the final flowering stage (**A**) and podding stage (**B**), respectively. Pathways directly or highly associated with lodging traits are highlighted in red, those indirectly associated are shown in green, and those with no significant association are displayed in black.

KEGG pathway analysis of DEGs from stem comparisons between GY and TY at the final flowering and podding stages revealed a complementary transcriptional strategy that underlies enhanced lodging resistance (Figure 6). At the final flowering stage, among the top 20 significantly enriched metabolic pathways, two showed no association with lodging, thirteen were indirectly associated, and five were directly or highly associated with the trait (Figure 6A). These indirectly involved in lodging resistance pathways include primary metabolic processes such as galactose metabolism and starch and sucrose metabolism, which supply essential precursors for cell wall biosynthesis and serve as prerequisites for the formation of robust stems. The five pathways directly or strongly correlated with lodging resistance function through two primary mechanisms: those reinforcing cell wall strength via the deposition of lignin, hemicellulose, and pectin (phenylalanine metabolism, phenylpropanoid biosynthesis, galactose metabolism), and those coordinating growth and defense by transducing hormonal and environmental signals (plant hormone signal transduction, MAPK signaling pathway).

During the podding stage, ten of the top 20 enriched pathways were indirectly linked to lodging resistance and are marked in green in Figure 6B. Primary metabolic pathways such as fructose and mannose metabolism were included, supporting lodging-resistant traits through the provision of precursors for cell wall assembly. Three pathways shown in red were directly associated with lodging resistance (Figure 6B). Among these, phenylpropanoid biosynthesis and cutin, suberine, and wax biosynthesis are secondary metabolic pathways strongly tied to stem strengthening. In addition, fructose and mannose metabolism, although categorized as a primary metabolic pathway, directly supplies key substrates for cell wall polysaccharides such as pectin and hemicellulose, thereby influencing the mechanical properties of the cell wall.

### 2.6. DEGs Related to Phenylpropanoid Biosynthesis Pathway

**Figure 7:**
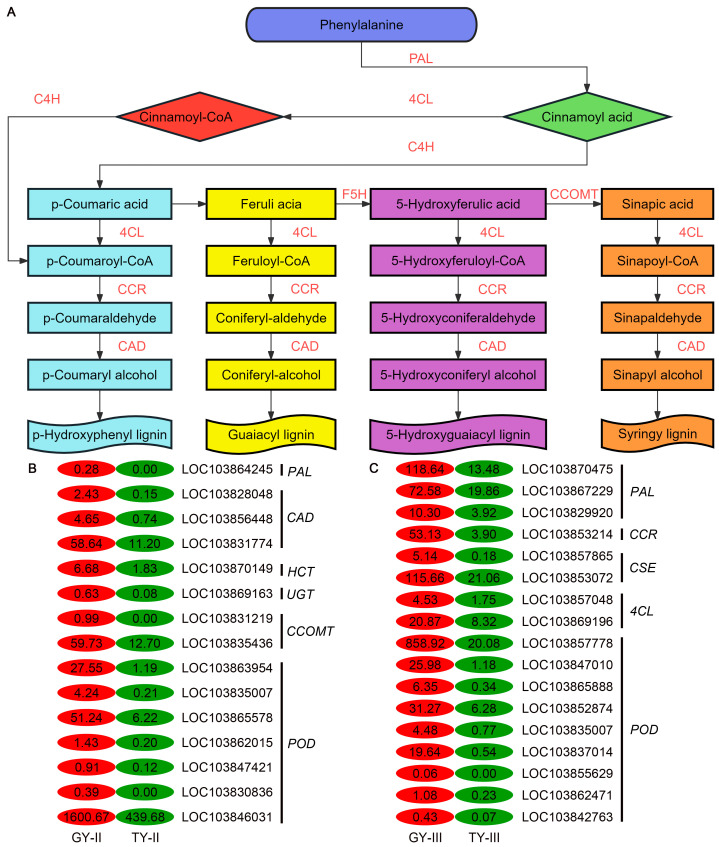
Expression patterns of lignin biosynthetic genes in two rapeseed genotypes with contrasting lodging resistance. (**A**) A schematic representation of the core phenylpropanoid pathway leading to lignin synthesis. (**B**,**C**) Heatmap depicting DEGs related to lignin biosynthesis. The color scale reflects gene expression levels, with red indicating significantly higher and green indicating significantly lower expression in the resistant genotype (GY) compared to the susceptible one (TY).

Lignin is deposited within the cellulose framework to enhance mechanical strength, facilitate water transport, and improve resistance against biotic and abiotic stresses. The phenylpropanoid biosynthesis pathway is essential for lignin production (Figure 7A). In this pathway, phenylalanine is first converted to cinnamic acid by phenylalanine ammonia-lyase (PAL), and then to p-coumaric acid by cinnamate 4-hydroxylase (C4H), a key branching point in phenylpropanoid metabolism. p-Coumaric acid is subsequently channeled through a series of enzymatic steps involving 4-coumarate:CoA ligase (4CL), cinnamoyl-CoA reductase (CCR), and cinnamyl alcohol dehydrogenase (CAD), along with hydroxylation and methylation reactions, to generate various monolignol precursors. These precursors are ultimately polymerized into different types of lignin via oxidative coupling.

In the phenylpropanoid biosynthesis pathway, a total of 48 DEGs were identified at the final flowering stage, comprising 18 upregulated and 30 downregulated genes. The upregulated DEGs included one PAL, three CADs, two caffeoyl-CoA O-methyltransferases (CCoAOMTs), one shikimate O-hydroxycinnamoyltransferase (HCT), one UDP-glycosyltransferase (UGT), and seven peroxidases (PODs) (Figure 7B). During the podding stage, 57 DEGs were detected, of which 19 were upregulated and 38 were downregulated. The upregulated genes encompassed three PALs, one cinnamoyl-CoA reductase (CCR), two caffeoyl shikimate esterases (CSEs), two 4CLs, and nine PODs (Figure 7C). Heatmap analysis revealed that the expression levels of these lignin-related genes were significantly higher in the lodging-resistant genotype GY than in the susceptible genotype TY, consistent with the observed lignin content (Figure 7B,C). Quantitative real-time PCR (qPCR) analysis was performed to validate the expression patterns of key lignin biosynthesis genes (*PAL3*, *CCoAOMT*, *POD*) and the cell wall remodeling gene *XTH1*. The results demonstrated that these genes were significantly upregulated in the lodging-resistant line (Figure S1). Furthermore, their expression levels were notably higher at the final flowering stage than at the podding stage. This spatiotemporal expression pattern showed a strong concordance with our transcriptomic data, with a coefficient of determination (R^2^) exceeding 0.9 (Figure 4C). As illustrated in Figure 7A, the upregulation of these genes promotes the formation of lignin monomers. The enhanced expression of lignin biosynthetic genes in GY likely contributes to the higher lignin accumulation and consequently improved lodging resistance observed in this genotype.

### 2.7. Variations in Stem Cell Wall Composition and Microstructure

**Figure 8:**
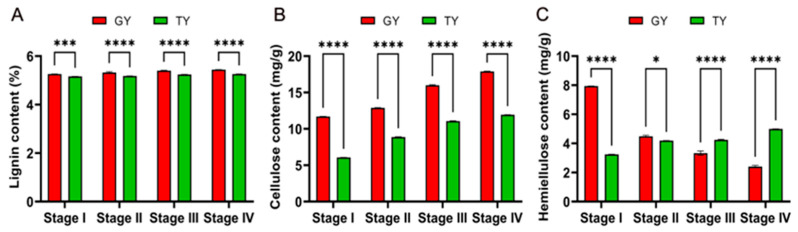
Stem cell wall composition. Lignin content (**A**), cellulose content (**B**) and hemicellulose content (**C**), respectively. Data are presented as mean ± SD (*n* = 3). Two-way ANOVA analysis was used. Asterisks denote significance levels: * *p* < 0.05, *** *p* < 0.001, and **** *p* < 0.0001.

As shown in Figure 8, significant differences were observed in the contents of the major cell wall components between the two cultivars across all four growth stages. Specifically, GY exhibited significantly higher lignin and cellulose contents than TY throughout the developmental period (Figure 8A). The most pronounced difference in cellulose content occurred at the full flowering stage, where GY showed a 1.93-fold increase compared to TY (Figure 8B). Regarding hemicellulose content, GY displayed a significant decreasing trend as development progressed, whereas TY exhibited an increasing trend (Figure 8C). This pattern in GY contrasted with its cellulose content dynamics, indicating a distinct regulatory pattern in cell wall composition between the two cultivars.

**Figure 9:**
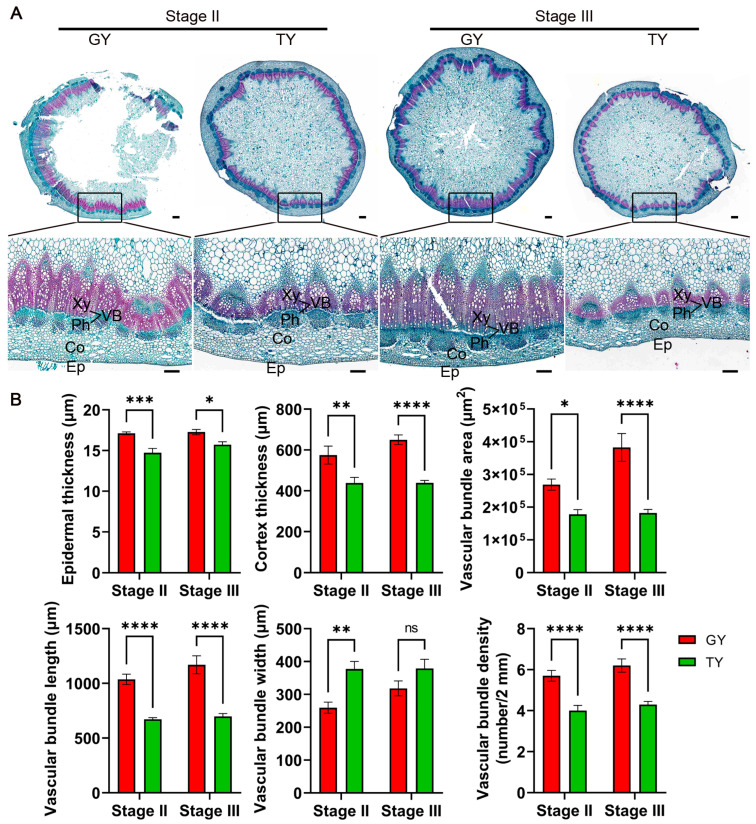
Stem microstructural phenotypes and data. (**A**) Representative cross-sectional images of stems from GY and TY at the final flowering (Stage II) and podding (Stage III) stages. Xy, xylem; Ph, phloem; VB, vascular bundle; Co, cortex; Ep, epidermis. Scale bar = 2 mm. (**B**) Quantitative data of microstructure: epidermal thickness, cortex thickness, vascular bundle area, length, width, and density. Values are presented as mean ± SD (*n* = 10). Two-way ANOVA analysis was used. Asterisks denote significance levels: * *p* < 0.05, ** *p* < 0.01, *** *p* < 0.001, and **** *p* < 0.0001. “ns” denotes no significant difference.

An investigation into the stem anatomy unveiled a consistently superior anatomical profile in GY during the final flowering and podding stages (Figure 9A). Compared to TY, GY demonstrated significantly increased epidermal thickness, cortical thickness, vascular bundle length, vascular bundle area, and vascular bundle density, though with a reduction in vascular bundle width (Figure 9B). These findings strongly suggest that the reinforced anatomical structure of GY contributes to its greater mechanical strength, thereby enhancing its lodging resistance.

## 3. Discussion

As an indispensable global oilseed crop, rapeseed faces a significant production constraint: lodging. Defined as the permanent bending or breaking of stems, lodging substantially compromises rapeseed yield by reducing photosynthetic efficiency and increasing susceptibility to disease, leading to considerable yield losses ranging from 20% to 50% [2,17,27]. This complex agronomic trait is governed by multiple interrelated factors, including stem mechanical strength, root anchorage capability, and plant architectural characteristics such as plant height and branching pattern [28,29,30]. The detrimental impact of lodging extends beyond yield, impairing both yield stability and quality, limiting biomass accumulation, and consequently restricting the industrial utilization of rapeseed as a raw material [2,21]. To systematically elucidate the mechanisms underlying lodging resistance, this study conducted a comprehensive investigation of two rapeseed cultivars, GY and TY, by integrating phenotypic, agronomic, anatomical, and transcriptomic analyses. Our findings demonstrate that the superior lodging resistance of cultivar GY is not attributable to a single factor, but rather stems from a synergistic interplay among optimal plant architecture, robust root system development, enhanced stem mechanical strength, and a unique transcriptional regulatory network.

A key finding of this study is that the similar final yield per plant between GY and TY was achieved through divergent strategies: GY relied on a higher silique number, whereas TY depended on a greater thousand-seed weight (Figure 1). This indicates that lodging resistance in GY does not come at the cost of yield, making it an ideal candidate for production. The critical agronomic traits associated with lodging, including a lower center of gravity, lower first branch height, and significantly higher stem breaking resistance, were consistently more favorable in GY across growth stages (Figure 2). These traits collectively contribute to a lower lodging index, effectively reducing the leverage force acting on the stem base and enhancing the plant’s ability to withstand external pressures. Our phenotypic findings align with and extend previous reports in other crops. For instance, the association between shorter stature, stronger stems, and lodging resistance is well-documented in wheat. Furthermore, the biomass analysis revealed a crucial distinction: while above-ground fresh weight was similar, GY accumulated significantly more dry matter in its aerial parts than TY during later stages (Figure 3A). Concurrently, GY exhibited lower stem water content. Since high water content is often correlated with reduced tissue strength and increased lodging risk, the higher dry mass and lower hydration state of GY stems likely contribute directly to their greater mechanical rigidity and resistance to breaking. The content of lignin and cellulose is an important influencing factor on the mechanical strength of canola stems, which is similar in different crops [31,32]. Stem mechanical performance is directly influenced by anatomical and compositional traits [11]. The GY cultivar demonstrated superior values over TY in key chemical components, namely lignin and cellulose content, as well as in anatomical features such as epidermal thickness and vascular bundle metrics (including number, length, density, and area) (Figure 8 and Figure 9).

Going beyond phenotypical assessments of stem biomechanics, physiology, and vascular anatomy, this study employed RNA-Seq on the basal 15 cm stem sections of two rapeseed cultivars with distinct lodging resistance. This specific region was targeted as it is the most prone to bending (Figure 1A). The content of lignin is associated with stem rigidity because lignin is only deposited in the secondary cell wall, which influences the structural integrity of the cell wall and stiffness of the stem [33]. The transcriptomic analysis offers a molecular explanation for these phenotypic advantages. The consistent enrichment of the phenylpropanoid biosynthesis pathway in resistant materials is a cornerstone finding (Figure 6 and Figure 7). The coordinated upregulation of genes in this pathway provides a direct genetic basis for the observed increase in lignin deposition and the consequent enhancement in stem mechanical properties and microscopic wall thickness (Figure 8 and Figure 9).

KEGG analysis between lodging-resistant and lodging-susceptible materials revealed systematic gene expression differences before (at the final flowering stage) and during (at the podding stage) lodging occurrence, uncovering a “predictive defense” biological mechanism (Figure 6). At the final flowering stage, the lodging-resistant material had already pre-activated defense preparations, with significantly enriched pathways including plant hormone signal transduction and the MAPK signaling pathway, demonstrating enhanced signal perception and transduction capacity (Figure 6A). Concurrently, the initiation of phenylpropanoid biosynthesis and related metabolic pathways supplied precursor compounds for lignin synthesis to reinforce the cell wall (Figure 6A). Upon entering the podding stage, the response strategy of the lodging-resistant material shifted towards execution and maintenance: the significance of the photosynthesis and photosynthesis-antenna proteins pathways markedly increased, ensuring the energy supply required for resistance; the active ribosome and DNA replication pathways sustained protein synthesis and cellular repair capabilities; while the continued activity of phenylpropanoid biosynthesis and glutathione metabolism pathways reinforced cell wall structure and antioxidant defense, respectively (Figure 6B). In contrast, the lodging-susceptible material lacked this coordinated transition from “signaling preparation” to “functional execution.” Its defense pathways were insufficiently activated at the final flowering stage, and it failed to maintain key physiological functions during the podding stage, ultimately resulting in inadequate mechanical tissue strength and lodging occurrence. This study elucidates the dynamic regulatory program underlying lodging resistance from a spatiotemporal perspective, providing a critical theoretical foundation for the identification of core genes.

Lodging is a complex agronomic trait that is strongly influenced by interannual climatic variations, such as rainfall patterns, wind velocity, and temperature fluctuations, as well as regional soil properties and ecological conditions. Consequently, inconsistent phenotypic performance might be observed across different eco-environments. However, our field experiments were conducted only for a single growing season at a single location. Furthermore, our investigations were restricted to just two *Brassica rapa* cultivars with contrasting lodging resistance (GY and TY). These two aspects impose certain limitations. Nevertheless, GY, as a lodging-resistant line bred by our team over multiple years, serves as the control counterpart to TY. Extensive field observations over years have consistently demonstrated that GY exhibits superior lodging resistance compared with TY. Although our data are limited to a single growing season at a single location, they effectively capture the distinct differences in lodging resistance between the two genotypes. We acknowledge that multi-year field trials are essential to confirm the stability of lodging-resistant phenotypes and to dissect genotype-by-environment interactions. Future studies will focus on verifying the functional roles of the candidate genes (*PAL3*, *CCoAOMT*, and *CAD9*) identified in this study across multiple environments, years, eco-regions, and a broader range of germplasm resources.

## 4. Materials and Methods

### 4.1. Plant Materials and Field Experiment Design

Two Brassica rapa materials were used in this experiment, including “Ganyou 3064” (a lodging-resistant cultivar, GY) and “Tianyou 2022” (a lodging-susceptible cultivar, TY). The seeds were preserved by the State Key Laboratory of Aridland Crop Science, Gansu Agricultural University. These materials were sown on 1 September 2023, in Zhangjiachuan, Tianshui City, Gansu Province. The planting maintained a row spacing of 35 cm and a plant spacing of 15 cm, with each cultivar occupying one mu (approximately 667 square meters) of experimental area.

We sampled at four growth stages: the full flowering stage (Stage I), final flowering stage (Stage II), podding stage (Stage III), and maturity stage (Stage IV). Before sampling at each growth stage, we first comprehensively observed and recorded the overall growth status and lodging tendency of the entire experimental populations of the two genotypes. Ten representative plants consistent with the overall performance of the population were randomly selected, ensuring that the selected samples were evenly distributed in the experimental plot and could truly reflect the overall phenotypic characteristics and lodging performance of the corresponding genotype at that growth stage.

### 4.2. Evaluation of Agronomic Traits and Lodging Resistance

The assessed morphological traits included plant height, gravity center height, first branching height (distance from the root collar), number of branches, number of siliques, number of seeds per silique, thousand-seed weight, yield per plant, and stem diameter. Stem diameter was measured at 15 cm above the root collar. Mechanical properties were characterized by applying a vertical downward force with a force gauge at the same position until stem fracture occurred. The maximum force recorded was designated as the stem breaking strength, while the corresponding vertical displacement of the force application point was quantified as stem flexibility [22]. Furthermore, the lodging coefficient (ILC) was calculated according to reference using the formula: ILC (g·cm·N^−1^) = shoot fresh weight (g) × gravity center height (cm)/stem breaking strength (N).

### 4.3. Determination of Biomass and Water Content

At the four aforementioned growth stages, the following determinations were performed on the ten randomly selected plants. The fresh weights of both aerial and root tissues were measured immediately after harvest and washing with deionized water, respectively. All tissues were then oven-dried at 80 °C to a constant weight to determine their dry weights. The following parameters were calculated: Moisture content (%) = [(Fresh weight − Dry weight)/Fresh weight] × 100%, for both aerial and underground tissues. The root crown ratio was calculated as the underground fresh weight divided by the aerial fresh weight.

### 4.4. Analysis of Root System Architecture

The root system architecture of the ten selected plants was characterized at the four aforementioned growth stages, with key parameters including root length (measured from the main root base to its tip using a ruler), root diameter (determined at the mid-section of the main root with a vernier caliper), and the number of lateral roots (counted as the total emerging from the main root).

### 4.5. Analysis of Stem Chemical Composition

The quantification of lignin, cellulose, and hemicellulose contents in the stem samples was performed using established analytical methods. Specifically, the lignin content was determined by the acetyl bromide method, which offers high reproducibility and sensitivity in detecting lignin in plant cell walls, as described in reference [34]. Cellulose content was assessed via the anthrone-sulfuric acid colorimetric method, a widely used approach based on the reaction of cellulose-derived sugars with anthrone reagent to produce a measurable color intensity [35]. Meanwhile, hemicellulose content was evaluated using the hydrochloric acid hydrolysis method, which facilitates the breakdown of hemicellulose into monomeric sugars for subsequent quantification, following the procedure outlined in reference [36].

### 4.6. Observation of Stem Microstructure

At the final flowering stage and the podding stage, stem segments approximately 1 cm in length were collected from a position 20 cm above the root crown using a sharp blade. The collected stem tissues were immediately fixed in 70% FAA solution (Formalin-Acetic acid-Alcohol) for a minimum of 48 h. Following fixation, the specimens were subjected to a series of graded ethanol solutions for dehydration. Subsequently, the dehydrated samples were embedded and sectioned. Transverse sections with a thickness of 10 µm were obtained and stained using a dual staining protocol. The sections were initially immersed in a 1% Safranin O solution for one hour, followed by dehydration and destaining in an ethanol series. They were then counterstained with a Fast Green solution for one hour. After a final dehydration series, the stained sections were mounted and observed under a digital microscope for imaging and morphological analysis [37].

### 4.7. RNA Extraction, Library Preparation and Transcriptome Sequencing

At the final flowering and podding stages, about 1 cm stem segments located 10–20 cm above the root crown were collected from both lodging-resistant (GY) and lodging-susceptible (TY) rapeseed genotypes. Total RNA was extracted using TRIzol reagent (Invitrogen, Carlsbad, CA, USA) following the manufacturer’s protocol. RNA integrity was verified by 1.2% agarose gel electrophoresis, and purity was assessed using a NanoDrop spectrophotometer (Thermo Scientific, Waltham, MA, USA). Samples with an A260/A280 ratio between 1.8 and 2.1 and an RNA Integrity Number (RIN) ≥ 7.0, as determined by an Agilent 2100 Bioanalyzer, were used for downstream analyses.

Sequencing libraries were constructed using the NEBNext Ultra RNA Library Prep Kit (New England Biolabs Inc.; Ipswich, MA, USA) for Illumina. Briefly, mRNA was enriched from total RNA using Oligo (dT) magnetic (Thermo Fisher Scientific, Waltham, MA, USA) beads and fragmented via divalent cations under elevated temperature. First- and second-strand cDNA were synthesized, followed by end repair, adenylation, and ligation of Illumina-indexed adapters. cDNA fragments of approximately 400–500 bp were selected using AMPure XP beads (Beckman Coulter, Beverly Hills, CA, USA), amplified by PCR, and repurified to construct the final sequencing library. Library quality was evaluated using the Agilent High Sensitivity DNA Kit (Agilent Technologies Inc., Santa Clara, CA, USA), and quantification was determined using the PicoGreen fluorescence-based method (Quantifluor-ST fluorometer, Promega, Madison, WI, USA; Quant-iT PicoGreen dsDNA Assay Kit, Invitrogen, Carlsbad, CA, USA), and effective library concentration was accurately quantified via qPCR on a StepOnePlus Real-Time PCR System (Thermo Scientific, Waltham, MA, USA). Finally, multiplexed libraries were normalized, pooled in equal volumes, and sequenced on an Illumina platform in paired-end 150 bp (PE150) mode.

### 4.8. Go and KEGG Analysis

Differentially expressed genes (DEGs) were identified using DESeq2 (version 1.38.3), applying thresholds of |log_2_FoldChange| > 1 and an adjusted *p*-value < 0.05. The functional profiles of these DEGs were then interrogated through Gene Ontology (GO) and Kyoto Encyclopedia of Genes and Genomes (KEGG) pathway enrichment analyses implemented in the clusterProfiler package (version 4.6.0). Significance of enrichment was assessed via a hypergeometric test (*p*-value < 0.05). To gain nuanced biological insights, this functional enrichment was analyzed separately for the full set of DEGs, as well as for the up-regulated and down-regulated subsets, thereby elucidating the key processes, functions, and pathways underlying the transcriptional response.

### 4.9. Quantitative Real-Time PCR (qPCR) Validation

To validate transcriptomic results, cDNA was synthesized from 1 μg of total RNA using the PrimeScript RT Reagent Kit (Takara, Shanghai, China). Gene-specific primers were designed using Primer-BLAST (https://www.ncbi.nlm.nih.gov/tools/primer-blast/) (Table S1), and qPCR was performed in triplicate on a CFX96 Touch Real-Time PCR System (Bio-Rad, Shanghai, China) using SYBR Green Premix (Takara, Shanghai, China). The housekeeping gene *BrACTIN* was used as an internal control. Relative expression levels were calculated using the 2^−ΔΔCt^ method.

### 4.10. Data Visualization and Statistical Analysis

All bar charts were generated using GraphPad Prism software (version 9.0). Data are presented as mean ± standard deviation (SD) with three or ten biological replicates (as described in the corresponding experimental details). One-way ANOVA (for comparisons between one group) or two-way ANOVA (for comparisons among multiple groups) was performed to determine significant differences between the lodging-resistant genotype GY and the lodging-susceptible genotype TY. Significant difference are indicated as follows: * *p* < 0.05, ** *p* < 0.01, *** *p* < 0.001, and **** *p* < 0.0001. “ns” denotes no significant difference. All images were assembled and combined using Adobe Photoshop CS4 (version 11.0.1).

## 5. Conclusions

**Figure 10:**
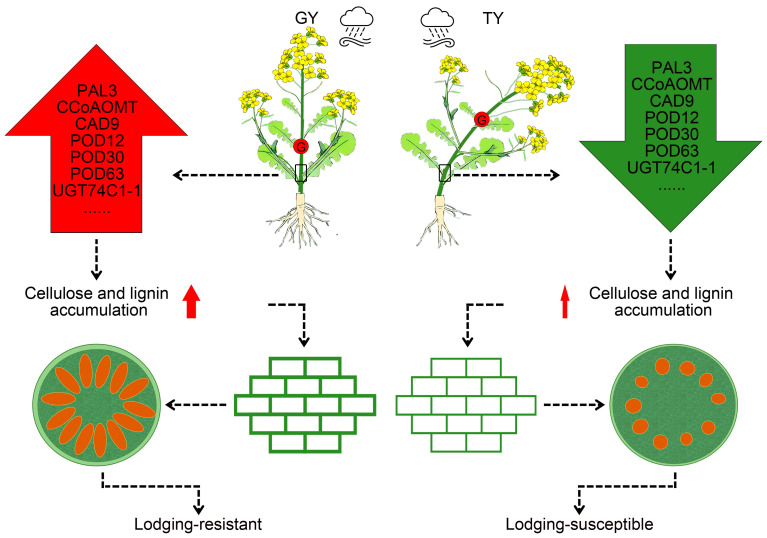
A proposed model illustrating the mechanistic basis of lodging resistance in rapeseed. Compared to the lodging-susceptible genotype TY, the lodging-resistant genotype GY exhibits a lower center of gravity and a more developed root system, which contribute to its overall mechanical stability. Concurrently, genes associated with lodging resistance, particularly those involved in lignin biosynthesis, are highly expressed in the stems. This elevated expression promotes the accumulation of cellulose and lignin, leading to increased cell wall thickness, as well as greater vascular bundle density and size. These structural enhancements collectively improve stem strength and support upright growth. In the schematic, ‘G’ denotes the center of gravity. Red upward arrows indicate up-regulated expression, while green downward arrows signify down-regulated expression. Arrows of different thicknesses represent the extent of lignin and cellulose accumulation. The red ovals in the stem cross-section represent the vascular bundles.

This study demonstrates that the superior lodging resistance of Gan You 3064 (GY) compared to Tian You 2022 (TY) arises from a synergistic interaction among plant architecture, anatomical structure, and transcriptional regulation (Figure 10). GY exhibited optimal phenotypic traits, including a lower center of gravity and a more robust root system, coupled with enhanced stem anatomical features such as thicker epidermal tissues and well-developed vascular bundles. At the physiological level, GY accumulated significantly higher levels of key cell wall components, particularly lignin and cellulose. Molecular analysis revealed that GY employs a preemptive transcriptional strategy, characterized by the early activation of the phenylpropanoid biosynthesis pathway and sustained upregulation of key lignin biosynthesis genes (e.g., *PAL*, *CAD*, *POD*) as early as the final flowering stage, thereby facilitating stem reinforcement prior to the lodging-sensitive podding stage. Traditional resistance mechanisms often depend on external stimuli or pressure signals to trigger defensive responses, which frequently lag behind the onset of damage. The coordinated improvement across morphological, physiological, and molecular levels collectively underpins the enhanced lodging resistance of GY, providing both theoretical insights and genetic resources for breeding lodging-resistant rapeseed varieties.

## Figures and Tables

**Table 1:** Analysis of the main components of rapeseed.

Component	GY	TY	Component	GY	TY
Protein	30.345 ± 0.461	30.074 ± 0.095	Linolenic acid	8.483 ± 0.271	8.388 ± 0.118
Oil content	35.563 ± 1.733	38.442 ± 0.222 *	Linoleic acid	15.601 ± 1.143	12.758 ± 0.165 *
Glucosinolate	67.340 ± 3.216	89.174 ± 3.529 *	Oleic acid	42.824 ± 1.609	29.524 ± 0.666 *
Palmitic acid	3.508 ± 0.286	2.754 ± 0.060	Erucic acid	23.119 ± 2.702	39.743 ± 0.424 *
Stearic acid	1.599 ± 0.0183	1.286 ± 0.029	Palmitoleic acid	0.206 ± 0.002	0.203 ± 0.002
Arachidic acid	0.646 ± 0.041	0.741 ± 0.006	cis-11-Eicosenoic acid	2.179 ± 1.146	3.867 ± 0.436
Behenic acid	0.593 ± 0.021	0.817 ± 0.022	11C,14C-Eicosadienoic acid	0.230 ± 0.007	0.297 ± 0.004

Data represent mean ± SD (*n* = 10). One-way analysis of variance (ANOVA) was used. Asterisk (*) indicates a significant difference between GY and TY at the *p* < 0.05 level.

## Data Availability

The original contributions presented in this study are included in the article.

## Supplementary Materials

The following supporting information can be downloaded at: https://www.mdpi.com/article/10.3390/plants15071134/s1, Figure S1: Quantitative real-time PCR (qPCR) analysis. Twenty selected genes were employed to validate the reliability of the transcriptome data; Table S1: Primers for qPCR analysis.
