# Supplementary material for: Comparing Transcriptome and Stem Anatomy Analysis Reveals That the Phenylpropanoid Pathway Is a Key Driving Factor for Lodging Resistance in Brassica rapa
Source: Plants (Basel). 2026 Apr 7;15(7):1134. doi: 10.3390/plants15071134 (PMC13074873; doi:10.3390/plants15071134)
Supplement: Supplementary file 1 [file plants-15-01134-s001.zip › plants-4209952-supplementary.pdf]

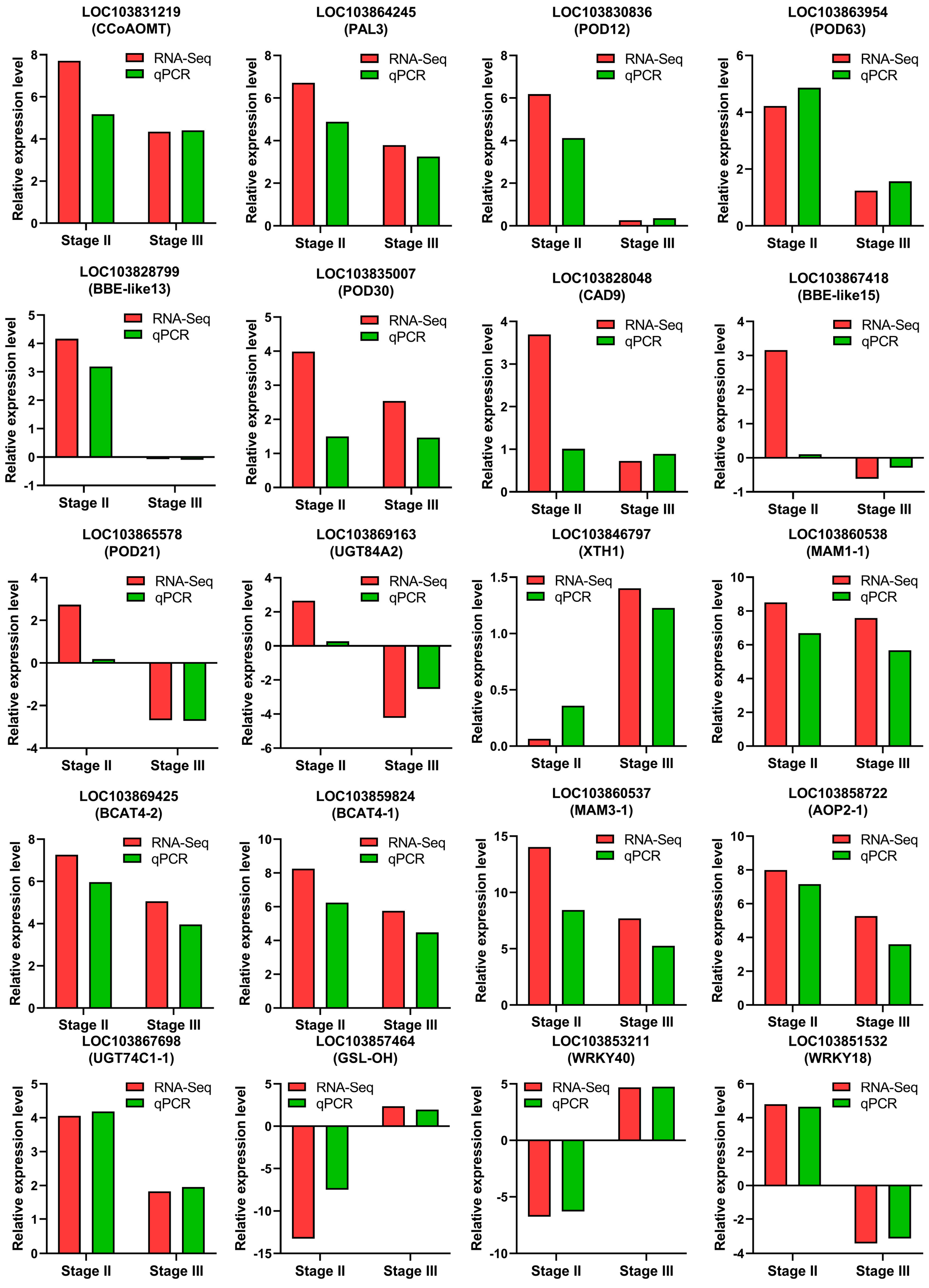

Figure S1. Quantitative real-time PCR (qPCR) analysis. Twenty selected genes were employed to validate the reliability of the transcriptome data.

Table S1 Primers for qPCR analysis.

	Forward	Reverse
LOC103846797	TCAATAATAGCCCTTGAAATATGAC	TGGATATGGCAGATGTGACG
LOC103860538	AATAAGGCTGGGACTGGTGC	AGTTCTTGTCGGGGAGCTTG
LOC103869425	GGAACGTGTTGTTCCCGTTG	CTTCTTATCCTCGACCCGGC

LOC103859824	AGGAACGTGTTGTTCCCGTT	CCGCCAATGTTGTATCCCCT
LOC103860537	CTCCCAGTTATATGCGCCGT	TCTTTGCGTACTTCACCGCT
LOC103858722	AGGAACTTGTGGACGAGCAG	CTCTACGGCCAACCTCAGTG
LOC103851532	AGATGAGAGCGCAAGTGAGT	GCTAGCGTCTCCGTTAGCTT
LOC103867698	AGTGGCCGGTGAAGAACATT	CAGACTCGTCAGGCTCAGTC
LOC103857464	CAACCCCTAAACCTCCCTCG	AACCTTCGCAACCACACTCT
LOC103853211	GTGGCCTAAACCGTTCTCCT	TCAAGGAAGACGCCATTTGC
LOC103831219	ATGGCGAATGAAATACCTACC	TCATACAAGGCGCCTGCAG
LOC103864245	TGGAATTTTGTCAACCAAAC	CGAAAAGAACGCTTCTCAGCTT
LOC103830836	ATGGCTAAGGGTTTATCTAAACGTATC	CAAAGCTCTCCACAATACCACG
LOC103863954	ATGGCAAAACTCATCATCCTCC	TTAGTTGATTGCATCACACCTTCG
LOC103828799	ATGTACATGAAACTCATCAAACAAAGAG	TCACTTAAATTTTGTAGGAATACTTTGTTC
LOC103835007	ATGAAGACGACGAATCGCTT	CTAACTTCGGGCAACAGAACAAC
LOC103828048	ATGCACCACTCCCTTGAGATAAC	TCATGGAGGGCTCAAGGAGT
LOC103867418	ATGGCGTTTGCGGTTTCA	TCAACCAATCTTTGTTGG
LOC103865578	ATGGCCATTGTGAAGATGCC	TTAGTTCACATAACGACAATCCTTCC
LOC103869163	ATGGAACTATCATCTTCTCCTTTACCT	TTATGACTTTTGCAATAAAAGTTTTCG
Actin	CATTGCAGATTGTTGCGAACAC	CCACTCCATCATCTTCATGG
